# Women’s experiences of maternity care in England: preliminary development of a standard measure

**DOI:** 10.1186/s12884-019-2284-9

**Published:** 2019-05-14

**Authors:** Maggie Redshaw, Colin R. Martin, Emily Savage-McGlynn, Sian Harrison

**Affiliations:** Policy Research Unit in Maternal Health and Care, National Perinatal Epidemiology Unit, Nuffield Department of Population Health, University of Oxford, Old Road Campus, Headington, Oxford, OX3 7LF UK

**Keywords:** Maternity, Patient experience, Psychometrics, Methodology, Perinatal, Birth, Pregnancy

## Abstract

**Background:**

As maternity services evolve and the population of women served also changes, there is a continuing need to effectively document the views of women with recent experience of care. A woman’s maternity experience can have a positive or negative effect upon her emotional well-being and health, in the immediate and the long-term, which can also impact the infant and the wider family system. Measuring women’s perceptions of maternity services is an important way of monitoring the quality of care provision, as well as providing key indicators to organisations of the services that they are providing. It follows that, without information identifying possible areas in need of improvement, it is not clear what changes should be made to improve the experiences of women during their journey through maternity services from pregnancy to the early weeks at home with a new baby .

The objective is to describe the development process and psychometric properties of a measure of women’s experience of maternity care covering the three distinctly different phases of maternity – pregnancy, labour and birth, and the early postnatal period.

**Methods:**

Data from a national survey of women who had recently given birth (*n* = 504) were used. Exploratory and confirmatory factor analytic methods were employed. The measure was assessed for underlying latent factor structure, as well as for reliability, internal consistency, and validity (predictive, convergent and discriminant).

**Results:**

The models developed confirmed the use of three separate, but related scales about experience of maternity care during pregnancy, labour and birth and the postnatal period. Data reduction was effective, resulting in a measure with 36 items (12 per scale).

**Conclusion:**

The need for a psychometrically robust and qualitatively comprehensive measure of women’s experience of maternity care has been addressed in the development and validation of this prototype measure. The whole measure can be used at one time point, or the three separate subscales used as individual measures of experience during particular phases of the maternity journey with identified factor structures in their own right.

**Electronic supplementary material:**

The online version of this article (10.1186/s12884-019-2284-9) contains supplementary material, which is available to authorized users.

## Background

For many women, the birth of a child is considered a major life event, particularly for those whom it marks the transition to parenthood. The experiences of being pregnant, giving birth, and the early days with their baby create memories that are likely to stay with them for a lifetime. Positive experiences during this time can be looked back upon fondly, empowering the woman in her role as a mother, and strengthening her emotionally during her transition to motherhood [1–4]. Conversely, a negative maternity experience may significantly increase the risk of negative health outcomes for the mother such as postnatal mental health disorders with possible long-lasting effects on the mother, the child, and the family system as a whole.

The care a woman receives during the perinatal period can have a profound impact on her overall maternity experience, with potentially significant implications for her health and wellbeing both at the time and subsequently [5–7]. In turn, this can impact on the mother-baby relationship and also on the health and wellbeing of the baby [8]. A woman’s experiences and memories of maternity care might also influence her decision-making regarding future pregnancies, requests for medical intervention during future childbirth, as well as having an impact on future reproduction in general. Thus, it is necessary to monitor, evaluate and optimise the care that women and their families receive during this important time.

From the perspective of policy, asking women about their views and experiences of care during the perinatal period is an effective way of assessing the quality of the maternity care received, and can provide key markers to healthcare providers at both specialty and organisational levels [9, 10]. As maternity services evolve and the population of women they serve changes, there is a continuing need to evaluate and document the views of women with recent experiences of care [11, 12].

The measurement of views and perceptions of any life-changing experience is challenging. In the context of an emotionally-charged, sometimes highly stressful, and physically demanding context such as pregnancy, childbirth, and the early postnatal period, effective measurement is particularly difficult. Women’s experience of maternity care is multidimensional, and its measurement must aim to account for an accurate representation of the many different aspects of care. Furthermore, there are multiple theoretical constructs involved in this area of healthcare – the attitudes and expectations held by women, the elements of choice in the available care options, differences in women’s needs, communication with women, information-giving by health professionals and perceptions of the care received. Perceptions of maternity care are also a function of the time period covered and nature of the pregnancy, birth and the associated events. There are thus difficulties inherent in trying to measure women’s overall experiences of maternity care with differing circumstances, expectations and needs during each of the different stages of the maternity journey.

While women may have variable experiences of maternity care and while the importance placed on each aspect of care may differ between women, there are notable themes of care that are known to be valued highly by women throughout the perinatal period. In particular, we know that women want to be informed and they value the opportunity for choice with respect to certain aspects of care, whether this is seeing a midwife at home or in her local surgery or choice of birth setting [13]. Respectful care is also valued by women, with privacy and dignity as the fundamental aspects for consideration. It has been shown that caring and respectful relationships with healthcare providers can significantly contribute to the overall birth experience [14, 15]. The quality of staff relationships, the strength of communication and the extent of continuity of care are other key elements that may contribute to women’s experiences [16, 17]. Having the opportunity to develop a rapport with her healthcare providers over the course of multiple antenatal appointments, or feeling understood and listened to by staff during labour are likely to contribute positively to a woman’s experience [5, 18–20].

It is perhaps the involvement of multiple elements in this area of healthcare and the diverse aspects that are differentially important to women at different time points during the perinatal period that has given rise to the multitude of measurement tools, which ask women about their experiences of maternity care in diverse ways. While some measures are purposely framed as being concerned with ‘satisfaction’ with care, this global commonly used term is difficult to define as it relates to care, being underpinned by multiple constructs that relate to the providers, the environment and the woman herself [21, 22]. Thus, satisfaction may relate to the overall experience of labour or birth, a woman’s own behaviour during the experience, or the care and treatment received from health care professionals or significant others [23]. Furthermore, it is possible to be satisfied with some aspects of an experience and dissatisfied with other aspects of that same experience [24]. There is also a lack of consistency in the way satisfaction has been measured and in the timing of the assessment [23, 25].

The organisations providing maternity care in the UK (NHS Trusts) have most often relied on the questionnaires developed and used by the Care Quality Commission (CQC) to rate trusts that are providing maternity care. These questionnaires allow performance to be compared across trusts and over time [26, 27] and organisations can also use the data to support local quality improvement [9, 10]. While these well-used surveys have originated to monitor women’s views and to audit the functioning of healthcare organisations [10, 11, 28], the survey instruments used are not validated measures and, arguably, are not sufficiently nuanced to address the richness of women’s experience during this important time in their lives. It should be noted that some components of other surveys have been linked with validation of specific measures [29–31].

Other tools available range from the single Friends and Family Test (FFT) [32] through structured questions about specific aspects of care, such as the Labour Agentry Scale (LAS) which focuses on control during childbirth [33, 34] or the Wijma Delivery Expectancy Questionnaire (WDEQ-A) which measures fear of childbirth [35, 36], to a range of instruments specifically targeting satisfaction with labour and birth. Measures of satisfaction include the Mackey Satisfaction with Childbirth Rating Scale (MCSRS) [37], the original and revised Birth Satisfaction Scale (BSS, BSS-R) [38, 39] and the recently developed short version of this measure [40], and the Labour and Delivery Satisfaction Index (LADSI) [41]. Some of these measures of satisfaction have been used to focus on specific aspects of care. For example, the BSS has been used in birth setting studies, and the LADSI used in a comparison of midwifery unit care with obstetric unit care [42]. More clinically focused studies have employed a variety of instruments, such as the MCSRS to measure satisfaction with birthing position in the late stages of labour [43] or the LAS to assess the use of water for pain relief [44].The measures almost entirely focus on labour and birth care, with little in the way of validated instruments measuring care during pregnancy and the postnatal period.

In order to assess the effectiveness of maternity care and the use of different care models and interventions, it has been emphasised that there is a need for some consistency in the way women’s experiences and perceptions, including satisfaction, are measured and reported for the purposes of benchmarking and quality improvement. There is thus a necessity for a psychometrically robust and qualitatively comprehensive measure of women’s experiences of maternity care to provide an accurate picture of women’s views, both positive and critical, and to effectively represent their experiences of the care they received during the perinatal period.

The aim of this study was to develop a valid and reliable self-report measure of the Experience of Maternity Care (EMC) that examines salient aspects of experience retrospectively related to (i) pregnancy (ii) labour and birth and (iii) the early postnatal period. The following research questions were addressed: 1) Are the three EMC scales uni-dimensional or multi-dimensional? 2) Do they demonstrate adequate internal consistency, divergent and convergent reliability and known-groups discriminant validity? The study also aimed to explore the possible use of the measure as a total score. This paper describes the development process and the psychometric properties of the prototype measure of experience of maternity care (EMC).

## Methods

### Measure development

#### Stage 1: literature review

A preliminary review of the literature was conducted to identify key themes relating to women’s experiences of care during the three phases of the perinatal period – pregnancy, labour and birth, and the early postnatal period. A further review of the literature relating to patient experiences within a more general healthcare environment was also undertaken. Analysis of structured and open text responses of more than ten thousand women participating in three large-scale national maternity surveys [28, 45, 46] were utilised to corroborate the findings of the literature search. Previously used measures were also reviewed [47]. The literature review identified a number of constructs that for theoretical reasons we wished to incorporate and make integral to the current measure: choice, control, access to care, perception of safety and wellbeing, continuity of care, information provision and communication by healthcare professionals [33, 48]

#### Stage 2: item generation

In preparation, a preliminary item generation phase was conducted with a goal of generating a minimum of 20 items per scale for the purposes of psychometric evaluation and final data reduction. A modular approach was taken. Items were generated specifically for each phase of maternity care (pregnancy, labour and birth, and postnatal care scales) so that they could be used as three distinct scales to look specifically at each phase of maternity, or as a ‘full-form’ to review the entire maternity experience. The scoring system chosen uses a five-point Likert approach ranging from ‘strongly agree’, ‘agree to some extent’, ‘neither agree or disagree’, ‘disagree to some extent’, and ‘strongly disagree’. To improve the administration experience and reduce repetition fatigue for participants, some questions were reverse worded and scored, with higher scores indicating a comparatively more positive experience of maternity care.

Examples of statements to which women could respond are: “*I felt I had the right number of antenatal checks with a midwife/doctor”*; “*Staff communicated well with me during labour and birth*” and “*After I had given birth, health professionals treated me as though I was no longer important*”.

#### Stage 3: cognitive interviews

Of crucial importance in the construction of the measure was the face validity for women of individual items and the overall scales. To further verify the acceptability of items and the overall conceptual framework of the measure, cognitive interviews were conducted with nine mothers who had recently given birth. The women were sent the questionnaire to complete in advance, and then interviewed to elicit their feedback and insight relating to individual items and overall content of the measure. During these sessions they were ‘thinking aloud’ about their responses, discussing the topics covered, and giving insight into the things they felt were important to be asked about their maternity experiences. Their feedback confirmed the items selected and informed the wording and ordering within the measure. It was also noted from the feedback that a full-form administration of the developed measure could potentially be a burden to participants if the measure was too long, hence, the decision was taken to limit the total number of items in the full-form version to a maximum of 36 items (12 per scale).

In requiring an initial stage of working with women directly ethical approval for the cognitive interviews was obtained from Oxford University Medical Sciences Interdivisional Research Ethics Committee (IDREC R46227/RE001) followed by a later application for the ONS managed national survey of infant and maternal health from the NRES committee for Yorkshire and The Humber – Sheffield (REC reference 16/YH/0412).

### Design and participants

The study on the measure utilised a two-stage cross-sectional design consistent with accepted instrument development practice [49–51]. Specifically, a random split-half data procedure was undertaken with the first split-half dataset (dataset one) used to determine underlying factor structure and item redundancy and the second split-half dataset (dataset two) to confirm factor structure and evaluate key psychometric properties of the measure.

The measure was a component of a larger postal survey with a cross-sectional design. Women (*N* = 2000) were selected randomly by the Office for National Statistics (ONS) from birth registrations in 2016. Stratification of the sample was based on births in different geographical areas (Government Office Regions). Women experiencing a perinatal loss and young mothers less than 16 years of age were excluded. In addition to the measure, the survey included questions relating to infant and maternal health, including infant feeding, return to work post-maternity, and maternal smoking during the perinatal period. The ONS mailed a letter of invitation, the questionnaire, an information sheet, a contact information sheet in multiple languages and a freepost return envelope to women at either three or 6 months postpartum. Women could complete the questionnaire on paper, online or verbally by telephone with a Language-Line interpreter if required. A tailored reminder system was used [52].

### Statistical analysis

#### Data preparation

The dataset questionnaire items were initially screened for accuracy, missing data, distributional normality and outliers. Kline [53] suggests skew values > 3 and kurtosis > 10 indicate non-normality. Missing value replacement in the event of missing data at < 5% was conducted using multivariate imputation by chained equations (MICE). In the event of > 5% missing data per scale, the individual participant data would be excluded from further analysis. Outlier detection and elimination was conducted by estimating the distance from the centroid (Mahalanobis distance) and calculating a threshold chi-square at a significance value of *p* > 0.001.

#### Exploratory factor analysis

Exploratory factor analysis (EFA) with maximum-likelihood estimation and oblimin rotation was used to determine factor structure and identify redundant items on each of the three EMC scales. Determining the number of meaningful factors to extract was conducted by parallel analysis using Mplus [54] and R [55] statistical software packages. A significant item-factor loading was set at a coefficient level of 0.30 to maximise identification of candidate factor items and a coefficient level of 0.50 set to indicate a significant item-factor loading, consistent with the method of Redshaw et al. with another perinatal measure [30]. Consistent with contemporary practice, cross-loading items were rejected.

#### Confirmatory factor analysis

The factor structure identified in EFA for each EMC scale was evaluated in dataset two using confirmatory factor analysis (CFA) [53, 56]. Multiple goodness of fit tests [57] were used to evaluate the models, these being the comparative fit index (CFI) values greater than 0.90 indicate an acceptable data fit and values of 0.95 and a good fit [58, 59], the root mean squared error of approximation (RMSEA) values of less than 0.05 indicate a good fit to the data [60]; the standardised root mean square residual (SRMR) values of less than 0.08 indicate acceptable model fit and 0.05 or less a good fit [49, 59, 61].

#### Divergent validity

Divergent validity was determined by correlating EMC scale and sub-scale scores with participant age. It was predicted that there would be no significant relationship between EMC scale and associated sub-scale scores and participant age.

#### Convergent validity

Convergent validity was determined by correlating EMC scale and sub-scale scores with a single Likert-scored [1–5] question asking level of agreement with the statement ‘I did not have enough choice about my care during pregnancy’. The question was reverse scored, thus higher scores indicate comparatively greater choice. It was predicted that there would be a significant correlation between EMC scale and associated sub-scale scores and the choice question score.

#### Known-groups discriminant validity

Known-groups discriminant validity was evaluated by examining score differences across a range of variables where it was anticipated group differences would be likely and based on previous literature. Ethnicity, defined as (i) white UK or (ii) Black or Minority Ethnic (BME) was used as differentiator variable for the pregnancy care appraisal sub-scale and the labour and birth sub-scale. Grouping based on parity, that is having given birth previously (Yes/No) was used to evaluate the pregnancy information sub-scale and delivery type (normal, vaginal birth without instruments/ non-normal, instrumental, including caesarean section) was used to evaluate the antenatal communication sub-scale. A single question ‘Asked about mental health at booking’ was used to evaluate the pregnancy continuity and antenatal checks sub-scales. The presence/absence of health problems with the baby was used to evaluate all three postnatal sub-scales. The selection of a broad range of discriminant variables was also chosen on the basis of reducing type I error by reducing the number of multiple-comparisons for each discriminant variable. Alpha was adjusted for multiple comparisons, thus criteria for significance for these differentiator variables was set to 0.02. The approach to this adjustment was based on balancing the potential for ameliorating the risk of type I error with a realistic and considered opportunity of detecting differences between groups where such differences may be evident. Since individual t-tests were being conducted, adjustment based on a post-hoc testing rationale (for example with analysis of variance across three or more groups) would be inappropriate. The approach was thus to divide conventional criterion for significance (0.05) by the number of sub-scales being evaluated (*N* = 3) and thus rounded to a probability criterion of *p* = 0.02. The advantage being that a more conservative criterion is specified cognisant with the pertinent study parameters, while not being overly conservative as would if a probability specification was based on the absolute number of comparisons.

#### Internal consistency

An internal consistency analysis of the EMC total and sub-scales was conducted to determine acceptability for clinical and research applications using Cronbach coefficient alpha with an alpha of 0.70 or greater being indicative of acceptable internal reliability [50, 56]. Statistical analysis was conducted using the statistical software package R [55, 62].

## Results

### Descriptive results

A response rate of 28% was achieved for the pilot survey. Additional file 1 shows the flow of participants whose data were used in the analyses. A total of 504 women made postal responses, returning usable data on the measure. Complete EMC data were available for analysis on 488 participants (~ 3% missing data). Elimination of multivariate outliers from complete data resulted in a dataset for use in the analyses of *N* = 449 (~ 8% outlier removal). The mean age of participants was 32.07 (SD 5.24) years. The average duration of pregnancy was 39.07 (SD 2.34) weeks. The majority (*N* = 433) of women (97%) had a single baby. The majority (*N* = 415, 92%) of women had their baby in hospital. Two-hundred and thirty-one women (51%) had their baby delivered in either a midwifery-led unit or birth centre.

The random split-half procedure produced an EFA dataset of *N* = 225 and a CFA dataset of *N* = 224.

The means, standard deviations, skew and kurtosis of dataset one are shown in Table 1 representing the pregnancy (EMC1–20), labour and childbirth (EMC21–40) and postnatal items (EMC41–60). Examination of skew and kurtosis characteristics suggested each item to have a univariate normal distribution (skew < 3, kurtosis < 10).

**Table 1:** Mean, standard deviation and distributional characteristics of pregnancy, labour and birth, and postnatal scale items in split-half exploratory factor analysis dataset (*N* = 225)

Item		Mean	SD	Min.	Max.	Skew	Kurtosis
*Pregnancy scale*							
**EMC1**	**I felt I had the right number of antenatal checks with a midwife/doctor**	3.29	1.03	0	4	−1.61	1.88
EMC2	I did not have enough choice about my care during pregnancy *(*e.g. *who you saw, where and when)*	2.50	1.33	0	4	−0.30	−1.19
**EMC3**	**My care provider(s) gave me all the information I needed**	3.21	1.02	0	4	−1.38	1.22
**EMC4**	**I always saw the same midwife/doctor for my antenatal checks**	2.17	1.60	0	4	−0.19	−1.60
EMC5	I was not able to contact my midwife or other health professional when I needed to	3.02	1.25	0	4	−1.00	−0.25
EMC6	I was not always treated with respect and kindness by health professionals	3.18	1.34	0	4	−1.42	0.51
**EMC7**	**Health professionals did not always talk to me in a way I could understand**	3.48	0.90	0	4	−1.78	2.44
**EMC8**	**Antenatal appointments were too short to discuss any concerns about my pregnancy**	3.27	1.07	0	4	−1.39	1.01
EMC9	I was able to speak to a health professional about any worries or sensitive issues.	3.27	1.08	0	4	−1.69	2.22
**EMC10**	**I was not involved enough in decisions about my antenatal care**	3.19	1.11	0	4	−1.09	−0.01
EMC11	I felt listened to when I talked to my care provider about my pregnancy and birth	3.21	1.07	0	4	−1.54	1.80
**EMC12**	**I was happy with the number of health professionals who cared for me during my pregnancy**	3.06	1.14	0	4	−1.10	0.23
**EMC13**	**I was not given enough explanations about the antenatal scans and tests**	3.16	1.15	0	4	−1.25	0.53
**EMC14**	**I was not given enough information to make decisions about my antenatal care**	3.26	1.09	0	4	−1.34	0.79
EMC15	Seeing different midwives/doctors for antenatal care did not matter to me	1.95	1.30	0	4	0.14	−1.17
**EMC16**	**I would have liked more antenatal checks and scans**	2.24	1.32	0	4	−0.14	−1.14
EMC17	During pregnancy I was given enough information about where I could give birth to my baby *(*e.g. *home, hospital, midwife unit)*	3.09	1.07	0	4	−1.01	0.06
EMC18	Health professionals always treated me as an individual	3.36	0.91	0	4	−1.72	3.01
**EMC19**	**During pregnancy, I did not feel well cared for by health professionals**	3.43	0.99	0	4	−1.92	3.07
**EMC20**	**Overall, I was very pleased with the care I received in pregnancy**	3.40	0.89	0	4	−1.86	3.73
*Labour and birth scale*							
EMC21	Before my labour and birth I was well informed by my midwife/doctor about what would happen	3.25	0.97	0	4	−1.39	1.46
**EMC22**	**Staff communicated well with me during labour and birth**	3.37	1.00	0	4	−1.72	2.27
EMC23	My choices for labour and birth were not always respected	3.21	1.15	0	4	−1.43	1.13
**EMC24**	**I needed more staff support during labour and birth**	3.16	1.23	0	4	−1.28	0.41
**EMC25**	**Everything was explained to me well during labour and birth**	3.29	0.99	0	4	−1.50	1.59
**EMC26**	**I was treated as an individual by staff**	3.50	0.85	0	4	−2.11	4.68
**EMC27**	**I was not involved enough in decisions about procedures that were carried out** ***(*** **e.g.** ***breaking waters, epidural, caesarean section)***	3.28	1.06	0	4	−1.37	1.00
**EMC28**	**Health professionals left me alone more than I would have liked**	3.08	1.22	0	4	−1.08	−0.13
**EMC29**	**I felt that my pain relief needs were not managed well**	3.07	1.27	0	4	−1.13	0.01
EMC30	I had already met the staff who looked after me during labour and birth	0.76	1.33	0	4	1.50	0.73
**EMC31**	**I felt safe in the labour and birth environment**	3.41	0.91	0	4	−1.73	2.59
EMC32	Staff did not listen to my requests in managing my labour and birth	3.33	1.07	0	4	−1.51	1.32
**EMC33**	**The staff could have done more to help me to feel in control of my labour and birth**	3.10	1.28	0	4	−1.11	−0.18
**EMC34**	**I had confidence and trust in the staff caring for me**	3.44	0.91	0	4	−1.82	2.87
EMC35	Health professionals helped make labour and birth a really positive experience	3.17	1.04	0	4	−1.27	1.03
EMC36	The personal care I received could have been better during labour and birth	3.00	1.31	0	4	−1.01	−0.39
**EMC37**	**I did not mind being looked after by midwives or doctors I had not met before**	3.00	1.08	0	4	−1.12	0.66
EMC38	The choices I wanted were not available to me	2.92	1.40	0	4	−0.87	− 0.74
EMC39	My labour and birth experience was not as I expected	2.00	1.44	0	4	0.10	−1.30
**EMC40**	**I had the best possible care during labour and birth**	3.37	0.95	0	4	−1.86	3.44
*Postnatal scale*							
**EMC41**	**I received enough care and attention from staff on the postnatal ward**	2.72	1.23	0	4	−0.71	−0.68
**EMC42**	**I stayed in hospital as long as I wanted after the birth**	2.78	1.35	0	4	−0.76	−0.74
EMC43	Staff on the postnatal ward did not respond when I needed them	2.76	1.29	0	4	−0.55	−1.11
EMC44	I was not able to make choices about the postnatal care of me and my baby	2.97	1.22	0	4	−0.81	− 0.62
**EMC45**	**I was treated as an individual by midwives/doctors after the birth**	3.10	1.16	0	4	−1.25	0.62
**EMC46**	**After I had given birth, health professionals treated me as though I was no longer important**	2.98	1.27	0	4	−0.96	−0.42
EMC47	I did not know the midwives I saw after bringing my baby home	1.74	1.59	0	4	0.37	−1.47
**EMC48**	**I had enough information from health professionals about how to care for my baby**	3.10	1.06	0	4	−1.21	0.81
EMC49	I felt comfortable speaking with healthcare professionals about how I was feeling	3.31	0.96	0	4	−1.61	2.27
EMC50	The health professionals I saw after the birth did not really listen to me	3.32	0.95	0	4	−1.45	1.61
EMC51	I would have liked to have seen midwives more after the birth	2.49	1.37	0	4	−0.45	−1.03
**EMC52**	**I was able to build a good relationship with the healthcare professional(s) I saw after coming home**	2.49	1.29	0	4	−0.36	−1.05
**EMC53**	**I was not given the advice and information I needed by health professionals after my baby was born**	3.18	1.02	0	4	−1.17	0.58
**EMC54**	**There was not enough time to talk over my concerns with health professionals**	3.17	1.10	0	4	−1.10	0.14
EMC55	The advice I received from healthcare professionals about caring for my baby was consistent	2.67	1.28	0	4	−0.77	−0.53
**EMC56**	**I had all the checks I needed after the birth**	3.13	1.15	0	4	−1.33	0.76
**EMC57**	**After the birth of my baby, I knew who to contact if I had questions or concerns**	3.38	0.94	0	4	−1.71	2.48
EMC58	As a mother of a new baby I did not feel cared for and supported enough by health professionals	3.25	1.01	0	4	−1.20	0.55
**EMC59**	**The postnatal care I received did not meet the needs of me and my baby**	3.19	1.13	0	4	−1.23	0.44
**EMC60**	**Overall I was very pleased with the quality of my postnatal care**	3.13	0.99	0	4	−1.17	0.81

Items in bold were retained for the 36 item measure

### Exploratory factor analysis

The findings of the parallel analysis are summarised in Table 2 for each scale. Scree plots are shown in Additional file 2. EFA’s for the EMC ***Pregnancy*** and ***Labour and Childbirth*** scales revealed that following the removal of non-loading and cross-loading items, one factor was associated with a single item loading. The EFA’s in these circumstances were therefore rerun as 5 and 2-factor models respectively. The ***Postnatal*** scale appeared to be miss-specified as a 4-factor model since each iteration of the EFA following item removal revealed increasing ambiguous factor structure. The model was rerun as a 3-factor model and produced a good fit to the data. The fit indices associated with each EMC scale are summarised in Table 3.

**Table 2:** Parallel analysis factor N determination for each EMC scale (*N* = 224)

EMC Scale	Parallel analysis N	EFA factors	Initial item N	Final item N
Pregnancy	6	5 (SI loading)	15	12
Labour and Childbirth	3	2 (SI loading)	15	12
Postnatal	4	3 (miss-spec)	17	12

**Table 3:** Exploratory factor analysis model fit statistics for each EMC scale (*N* = 224)

EMC Scale	χ^2^ (df)	*p*	RMSEA	RMSR	CFI	TLI
Pregnancy	47.43 [40]	0.20	0.03 (0.01–0.06)	0.03	0.99	0.98
Labour and Childbirth	154.62 (76)	< 0.01	0.07 (0.05–0.08)	0.04	0.96	0.94
Postnatal	195.22 (88)	< 0.01	0.08 (0.06–0.09)	0.04	0.94	0.91

Following review of EMC scale retained items, items with either the lowest item-factor loadings or the least theoretical cogency to the factor-domain identified within each scale were removed to reduce each scale to a total of 12 items. The mean, standard deviation and distributional characteristics of EMC sub-scale and total scores are summarised in Table 4.

**Table 4:** Mean, standard deviation and distributional characteristics of EMC sub-scales and total scale scores (*N* = 225)

Scale and sub-scale	Mean	SD	Min.	Max.	Skew	Kurtosis
*Sub-scale*						
Care appraisal (PR)	10.00	2.31	1	12	−1.34	1.43
Information (PR)	6.60	1.83	1	8	−1.05	−0.13
Antenatal communication (PR)	9.66	2.57	2	12	− 0.84	− 0.33
Continuity (PR)	5.10	2.41	0	8	−0.38	− 0.95
Antenatal checks (PR)	5.49	1.92	0	8	−0.39	−0.56
Care quality (LB)	22.77	5.45	0	28	−1.39	1.87
Care needs (LB)	14.70	5.15	0	20	−0.84	−0.19
Adequacy of care (PN)	11.50	4.13	0	16	−0.80	− 0.24
HP Communication^a^ (PN)	11.98	3.55	0	16	−0.82	0.11
Individualised care (PN)	12.72	3.20	0	16	−0.99	0.73
*Total scale scores*						
Pregnancy scale total score	36.84	8.29	12	48	−0.66	− 0.41
Labour & birth scale total score	37.47	9.96	0	48	−1.18	0.92
Postnatal scale total score	36.20	9.10	5	48	−0.83	0.08
Six-item scale total (PR)	19.65	4.43	3	24	−1.02	0.57

^a^*HP* Health Professional

### Confirmatory factor analysis

CFA was conducted on dataset two (*n* = 225) specifying the three EMC models identified by EFA with 12 items per scale. A single-factor version of this model was also evaluated to verify the suitability of the measure across the entirety of the maternity time period. Model fit estimations for the multidimensional models revealed generally acceptable fit across the range of fit indices. Unidimensional versions of each EMC model revealed a comparatively inferior fit to data. The χ^2^ differences test revealed each EMC multi-dimensional model to offer a statistically significant superior fit to data compared to the equivalent unidimensional model (*p* < 0.001). The model fit characteristics of each model evaluated are shown in Table 5.

**Table 5:** Confirmatory factor analysis model fit statistics for each EMC scale (N = 225) comparing multidimensional and unidimensional versions

Model	EMC Scale	Factor N	χ^2^ (df)	*p*	RMSEA	SRMR	CFI	TLI
1	Pregnancy	5	82.06 [44]	< 0.01	0.06 (0.04–0.08)	0.04	0.96	0.94
2	Labour and Childbirth	2	133.10 [53]	< 0.01	0.08 (0.07–0.10)	0.05	0.94	0.93
3	Postnatal	3	133.06 [51]	< 0.01	0.09 (0.07–0.10)	0.06	0.93	0.91
4	Pregnancy	1	143.84 [54]	< 0.01	0.09 (0.07–0.10)	0.06	0.90	0.88
5	Labour and Childbirth	1	149.36 [54]	< 0.01	0.09 (0.07–0.11)	0.05	0.93	0.92
6	Postnatal	1	283.51 [54]	< 0.01	0.14 (0.10–0.15)	0.08	0.80	0.76

All EMC scales and sub-scales were observed to be positively and statistically significantly correlated with the sole exception of no observed statistically significant relationship between Pregnancy sub-scale ‘Continuity’ and Postnatal sub-scale ‘Adequacy of care’. Pearson’s *r* correlations between EMC scales and sub-scales are summarised in Table 6.

**Table 6:** Pearson’s *r* correlations between EMC scale and sub-scale scores. *p* < 0.01 unless otherwise indicated

EMC Scale	PR.1	PR.2	PR.3	PR.4	PR.5	LB.1	LB.2	PN.1	PN.2	PN.3	Preg	Lab	Post
*Sub-scales*													
Care appraisal (PR.1)	1.00												
Information (PR.2)	0.62	1.00											
Antenatal communication (PR.3)	0.65	0.59	1.00										
Continuity (PR.4)	0.49	0.31	0.36	1.00									
Antenatal checks (PR.5)	0.47	0.38	0.39	0.24	1.00								
Care quality (LB.1)	0.45	0.34	0.39	*0.17*	0.35	1.00							
Care needs (LB.2)	0.32	0.33	0.37	**0.15**	0.28	0.76	1.00						
Adequacy of care (PN.1)	0.29	0.30	0.31	0.09*	*0.18*	0.34	0.33	1.00					
HP Communication* (PN.2)	0.45	0.44	0.48	0.28	0.34	0.44	0.44	0.46	1.00				
Individualised care (PN.2)	0.45	0.42	0.44	0.20	0.42	0.45	0.43	0.56	0.65	1.00			
*Totals*													
Pregnancy total	0.87	0.76	0.82	0.66	0.64	0.45	0.39	0.31	0.53	0.51	1.00		
Labour & birth total	0.41	0.36	0.40	*0.17*	0.33	0.94	0.94	0.36	0.47	0.47	0.45	1.00	
Postnatal total	0.46	0.45	0.48	0.22	0.36	0.49	0.47	0.83	0.83	0.86	0.53	0.51	1.00

* *p* = 0.17, Bold *p* = 0.02, Italic *p* = 0.01

### Internal consistency

Calculated Cronbach’s alpha of the EMC total and sub-scale scores are summarised in Table 7. EMC-LB total scale and all sub-scales and EMC-PN total scale and all sub-scales exceeded minimum alpha criteria of 0.70. However, while the EMC-PR total scale and the Care Appraisal sub-scale exceeded minimum alpha criteria, all four remaining EMC-PR sub-scales failed to reach alpha acceptability threshold.

**Table 7:** Cronbach’s alpha of each EMC sub-scale and scale with 95% confidence intervals (*N* = 225)

Scale	Sub-scale	N items	Alpha	Alpha (95% CI)
Pregnancy		12	0.847	0.82–0.88
	Care appraisal	3	0.798	0.75–0.84
	Information	2	0.672	0.59–0.76
	Antenatal comm.	3	0.662	0.59–0.74
	Continuity	2	0.588	0.49–0.69
	Antenatal checks	2	0.456	0.32–0.59
Labour and birth		12	0.904	0.89–0.92
	Care quality	7	0.867	0.84–0.89
	Care needs	5	0.811	0.77–0.85
Postnatal		12	0.873	0.85–0.90
	Adequacy of care	4	0.800	0.76–0.84
	HP Communication	4	0.775	0.72–0.82
	Individualised care	4	0.753	0.70–0.81

### Divergent validity

No significant correlation was observed between EMC scale and associated sub-scale scores and participant age. Inferential analysis is summarised in Table 8.

**Table 8:** Pearsons *r* correlations between EMC total and sub-scale scores and participant age

EMC Scale and sub-scale	*r*	95% CI	*p*
*Sub-scale scores*			
Care appraisal (PR)	−0.02	−0.15 - 0.11	0.73
Information (PR)	0.04	−0.09 - 0.17	0.55
Antenatal communication (PR)	−0.01	− 0.14 - 0.12	0.87
Continuity (PR)	−0.01	− 0.14 - 0.12	0.88
Antenatal checks (PR)	0.11	−0.02 - 0.24	0.10
Care quality (LB)	0.11	−0.02 - 0.24	0.09
Care needs (LB)	0.12	−0.01 - 0.25	0.08
Adequacy of care (PN)	0.05	−0.08 - 0.18	0.48
HP Communication (PN)	0.06	−0.07 - 0.19	0.38
Individualised care (PN)	0.09	−0.05 - 0.21	0.21
*Total scale scores*			
Pregnancy scale total score	0.02	−0.11 - 0.15	0.75
Labour & birth scale total score	0.12	−0.01 - 0.25	0.06
Postnatal scale total score	0.07	−0.06 - 0.20	0.27

### Convergent validity

Correlations between EMC scale and sub-scale scores and the choice question were all found to be positively and statistically significantly correlated (Table 9).

**Table 9:** Pearsons *r* correlations between EMC total and sub-scale scores and the choice about Pregnancy care question

EMC Scale and sub-scale	*r*	95% CI	*p*
*Sub-scale scores*			
Care appraisal (PR)	0.44	0.33–0.54	< 0.01
Information (PR)	0.40	0.29–0.51	< 0.01
Antenatal communication (PR)	0.44	0.33–0.54	< 0.01
Continuity (PR)	0.32	0.20–0.44	< 0.01
Antenatal checks (PR)	0.27	0.15–0.39	< 0.01
Care quality (LB)	0.25	0.12–0.37	< 0.01
Care needs (LB)	0.23	0.10–0.35	< 0.01
Adequacy of care (PN)	0.29	0.16–0.40	< 0.01
HP Communication (PN)	0.29	0.17–0.41	< 0.01
Individualised care (PN)	0.31	0.19–0.42	< 0.01
*Total scale scores*			
Pregnancy scale total score	0.50	0.40–0.60	< 0.01
Labour & birth scale total score	0.25	0.13–0.37	< 0.01
Postnatal scale total score	0.35	0.23–0.46	< 0.01

### Known-groups discriminant validity

The mean EMC total sub-scale scores as a function of discriminant variable categorisation are summarised in Table 10. Predicted significant differences between groups were observed in EMC ***Pregnancy*** sub-scales ‘information’ and ‘antenatal communication’, ***Labour and Birth*** sub-scales ‘care quality’ and ‘care needs’ and ***Postnatal*** sub-scales ‘health professional communication’ and ‘individualised care’. Effect sizes were observed to range between small to medium.

**Table 10:** Mean EMC Scale and sub-scale scores as a function of specific differentiator variables

Scale and sub-scale	Differentiator			*t*	*p*	95% CI	ES (*d*)	*d* 95% CI	ES Interpretation
*Sub-scale scores*									
Care appraisal (PR)	*Ethnicity (N = 169/56):*	White UK	BME						
		10.14 (2.26)	9.55 (2.43)	1.66	0.79	−0.11–1.29	0.26	− 0.05–0.56	Small
Information (PR)	*Previous birth (N = 110/115):*	‘No’	‘Yes’						
		6.35 (1.73)	6.84 (1.90)	1.98	0.05	−0.96– −0.01	0.27	0.01–0.53	Small
Antenatal communication (PR)	*Delivery type (N = 132/93):*	Normal	Non-normal						
		9.70 (2.53)	9.22 (2.58)	2.19	0.03	0.07–1.44	0.30	0.03–0.56	Small
Continuity (PR)	*Asked about mental health (N = 32/190):*	‘No’	‘Yes’						
		4.38 (2.92)	5.25 (2.30)	1.92	0.06	−1.78–0.02	0.37	−0.01–0.74	Small
Antenatal checks (PR)	*Asked about mental health (N = 32/190):*	‘No’	‘Yes’						
		5.09 (2.16)	5.67 (1.88)	1.29	0.19	−1.20–0.25	0.25	−0.13–0.62	Small
Care quality (LB)	*Ethnicity (N = 169/56):*	White UK	BME						
		23.30 (5.16)	21.20 (6.03)	2.53	0.01	0.46–3.74	0.39	0.08–0.70	Small
Care needs (LB)	Ethnicity (*N* = 169/56):	White UK	BME						
		15.17 (4.96)	13.29 (5.49)	2.39	0.02	0.33–3.43	0.37	0.06–0.67	Small
Adequacy of care (PN)	*Baby health problems (N = 196/27):*	‘No’	‘Yes’						
		11.67 (5.23)	10.11 (3.88)	1.85	0.07	−0.11–3.22	0.38	−0.03–0.78	Small
Health professional communication (PN)	*Baby health problems (N = 196/27):*	‘No’	‘Yes’						
		12.17 (4.10)	10.30 (3.41)	2.61	< 0.01	0.46–3.29	0.54	0.13–0.94	Medium
Individualised care (PN)	*Baby health problems (N = 196/27):*	‘No’	‘Yes’						
		12.91 (4.26)	11.07 (2.97)	2.84	< 0.01	0.56–3.12	0.58	0.18–0.99	Medium
*Total scale scores*									
Pregnancy scale total score	*Asked about mental health:*	‘No’	‘Yes’						
		35.00 (8.85)	37.25 (8.18)	1.42	0.16	−5.37–0.87	0.27	−0.11–0.65	Small
Labour & birth scale total score	*Ethnicity:*	White UK	BME						
		38.46 (9.51)	34.48 (10.76)	2.62	< 0.01	0.99–6.97	0.40	0.10–0.71	Small
Postnatal scale total score	*Baby health problems:*	‘No’	‘Yes’						
		36.76 (12.27)	31.48 (8.41)	2.87	< 0.01	1.65–8.90	0.59	0.18–1.00	Medium

## Discussion

Measures of patient care experiences can provide a direct metric of the effectiveness of this part of the maternity healthcare system [10] and it has been widely recognized that indicators and metrics that reflect patient satisfaction as well as the effectiveness of clinical care, costs and outcomes are essential [63–65]. Women’s experiences of care during the perinatal period is a subject of considerable importance, and in developing and validating a measure on the three different phases of care this study contributes to a growing body of work on this aspect of healthcare provision. The resulting instrument with three scales, each with 12 items scored on a five point scale, and different factor structures can be used for research and audit purposes.

In the process of development of the current measure, we were keenly aware of the needs of perinatal healthcare, to know, for example, what is important to women, what matters to them about their experience of maternity care along the whole pathway and what the key aspects are that should be measured. We were also aware of the practicalities of how the necessary data may be collected and reported to inform and improve the quality of care being provided locally and nationally.

Routine measurement of patient experiences of maternity care provides an important overview of the quality of care available to women and change over time [9, 12]. With increased pressures on the NHS in terms of funding, staffing levels, and increased patient usage, it is even more important to make use of good quality, robust metrics to provide insights and document the impact of changes in services. When considering maternity services and the measurement of women’s experiences, there are two perspectives to take into account in developing an outcome measure that will provide meaningful and psychometrically robust results – those of the women completing the measure, and those of the organisation administering the survey and, ultimately, making use of the results to review and improve the health services they are offering. The strengths and limitations of the study.

### Strengths and limitations

The main strength of the study lies in the use of structural equation modelling through the use of both exploratory and confirmatory factor analytic methods. The total scale scores of the separate scales which have clearly been validated as such can be used as stand-alone measures, or jointly as a profile measure for the overall perinatal experience of care. However, given the rather different factor structures for the separate scales reflecting the diverse range of events and experiences in the different phases of maternity care would argue against a global score. The simple structure of the measure, with three relatively short scales, with factor scores where appropriate, allows for use in comparing across individuals and groups, as well as within individuals and groups across time, enabling the identification of specific issues for women and care providers as operational objectives change [48].

A particular strength of this work is the face validity of the measure, having been developed with a social and psychological theoretical understanding, well as the insight and input from mothers directly through qualitative interview and earlier survey responses. It was a core element of the development process to include the lived experiences of women in designing the measure. The relatively short individual scales reference each phase of maternity care and provide an account of the woman’s experience that can help to inform both healthcare organisations and practitioners.

One limitation of this work is that the response rate to the postal questionnaire containing the prototype measure was low. The decline in response rates to postal questionnaires over recent decades is well documented [66] and, despite the growing literature reporting methods to halt this trend [67], response rates remain low. Although the overall response to the larger postal survey was low, there were sufficient responses to undertake the key analyses for the development of the measure. It also has to be acknowledged that respondents, in this case, recent mothers, may not be fully honest and critical of the care they received when asked to complete a questionnaire by the organisation which provided their care [47], though the independent nature of this survey may have mitigated against this. A specific limitation arises in relation to the measurement of experience of care during pregnancy. The relatively low alpha for the pregnancy subscale factors is likely to reflect the long time window covered and complexity of the range of events that can occur in pregnancy. A larger sample size may have enabled a more effective exploration of the pregnancy factors and a possible reduction in the number of these. Further use of the measure will inform this point.

Patient experiences can be measured in numerous ways, but in order to capture the full range of the multidimensional maternity experience in a metric that is easy to administer and summarise the results [22, 24, 68], we chose to develop an instrument that covers three different time frames: the antenatal period, labour and birth, and the early postnatal period. We acknowledge that the measurement of the multifaceted experience of pregnancy, birth and the early weeks with a new baby is rife with potential methodological issues. This is perhaps reflected in the paucity of measures that cover antenatal and postnatal care and the multiplicity that concern diverse aspects of labour and birth.

### Conclusion

For each of phase of the perinatal journey, the majority of women in the UK are under the care of health professionals within the National Health Service. The type of care that they receive differs across pregnancy, labour and birth and the Experience of Maternity Care measure with three individual scales was developed to address this. Documenting women’s diverse experience of care systematically with a measure such as the EMC can potentially benefit both the women being cared for and the healthcare service. Information arising from the use of the EMC offers valid and reliable metrics, supporting and informing potential drivers for change and quality improvement.

## Additional files

Additional file 1: **Appendix 1.** Flow of participants whose data were used in the analyses. (DOCX 40 kb)

Additional file 2: **Appendix 2.** Scree plots following parallel analysis of the pregnancy, labour and birth and postnatal sub-scales. (PDF 112 kb)

## Abbreviations

BME: Black or Minority Ethnic
BSS: Birth Satisfaction Scale
BSS-R: Birth Satisfaction Scale Revised
CFA: Confirmatory Factor Analysis
CFI: Comparative Fit Index
EFA: Exploratory Factor Analysis
EMC: Experience of maternity care
FFT: Friends and Family Test
LAS: Labour Agentry Scale
LB: Labour and Birth
MICE: Multivariate Imputation Chained Equations
MSCRS: Mackey Satisfaction with Childbirth Rating Scale
ONS: Office for National Statistics
PN: Postnatal
PR: Pregnancy
RMSEA: Root Mean Squared Error of Approximation
SRMR: Standardised Root Mean Square Residual
WDEQ: Wijma Delivery Expectancy Questionnaire
