# Supplementary material for: Women’s experiences of maternity care in England: preliminary development of a standard measure
Source: BMC Pregnancy Childbirth. 2019 May 14;19:167. doi: 10.1186/s12884-019-2284-9 (PMC6518811; doi:10.1186/s12884-019-2284-9)
Supplement: Supplementary file 1 — Appendix 1. Flow of participants whose data were used in the analyses. (DOCX 40 kb) [file 12884_2019_2284_MOESM1_ESM.docx]

Women identified from birth registration records and sent the questionnaire, n=2,000

Refusals / blank, duplicate, unusable returns, n=29

Unique complete & usable returns,
n=554 (27.7%)

Online returns,
n=48 (2.4%)

Telephone returns,
n=2 (0.1%)

Postal returns,
n=504 (25.2%)

Complete EMC data in postal returns,
n=488 (24.4%)

Elimination of multivariate outliers,
n=39

Dataset used in analyses,
n=449 (22.5%)

EFA dataset,
n=225

CFA dataset,
n=224

Figure 1 Flow of participants used in data analyses for the development of the EMC
