# Supplementary material for: Women’s experiences of maternity care in England: preliminary development of a standard measure
Source: BMC Pregnancy Childbirth. 2019 May 14;19:167. doi: 10.1186/s12884-019-2284-9 (PMC6518811; doi:10.1186/s12884-019-2284-9)
Supplement: Supplementary file 2 — Appendix 2. Scree plots following parallel analysis of the pregnancy, labour and birth and postnatal sub-scales. (PDF 112 kb) [file 12884_2019_2284_MOESM2_ESM.pdf]

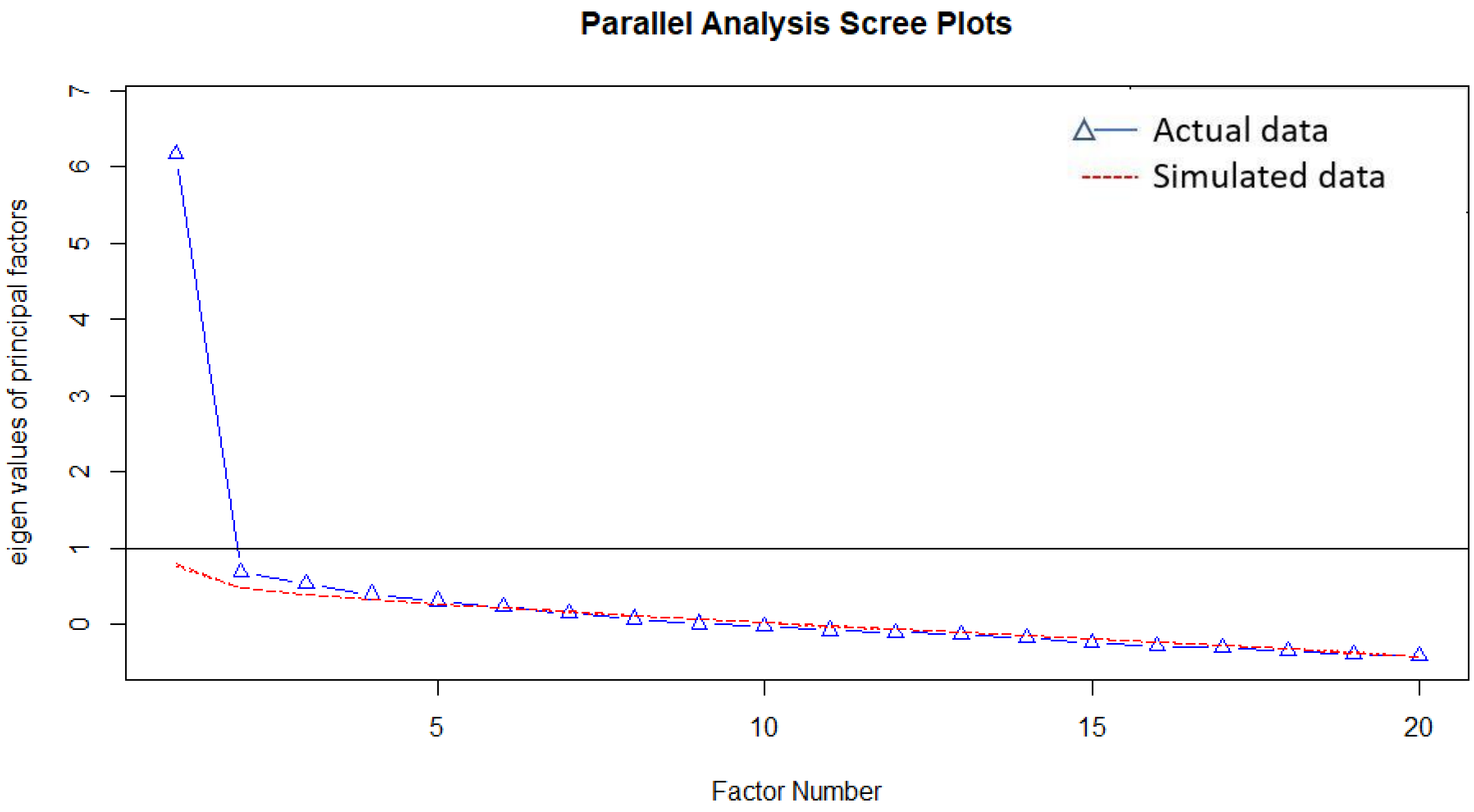

Figure 1. Scree plot following parallel analysis of the **pregnancy** scale.

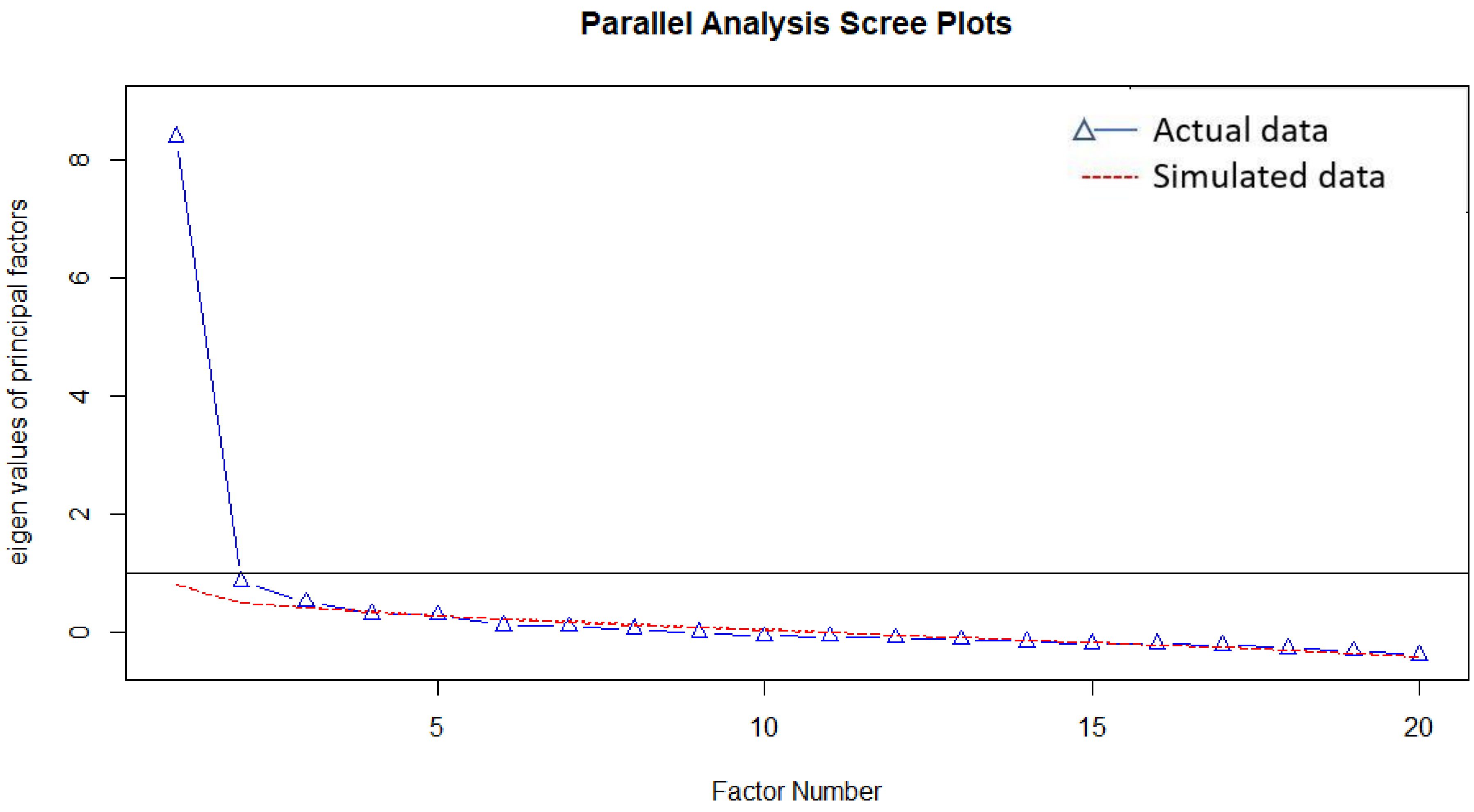

Figure 2. Scree plot following parallel analysis of the **labour and childbirth** scale.

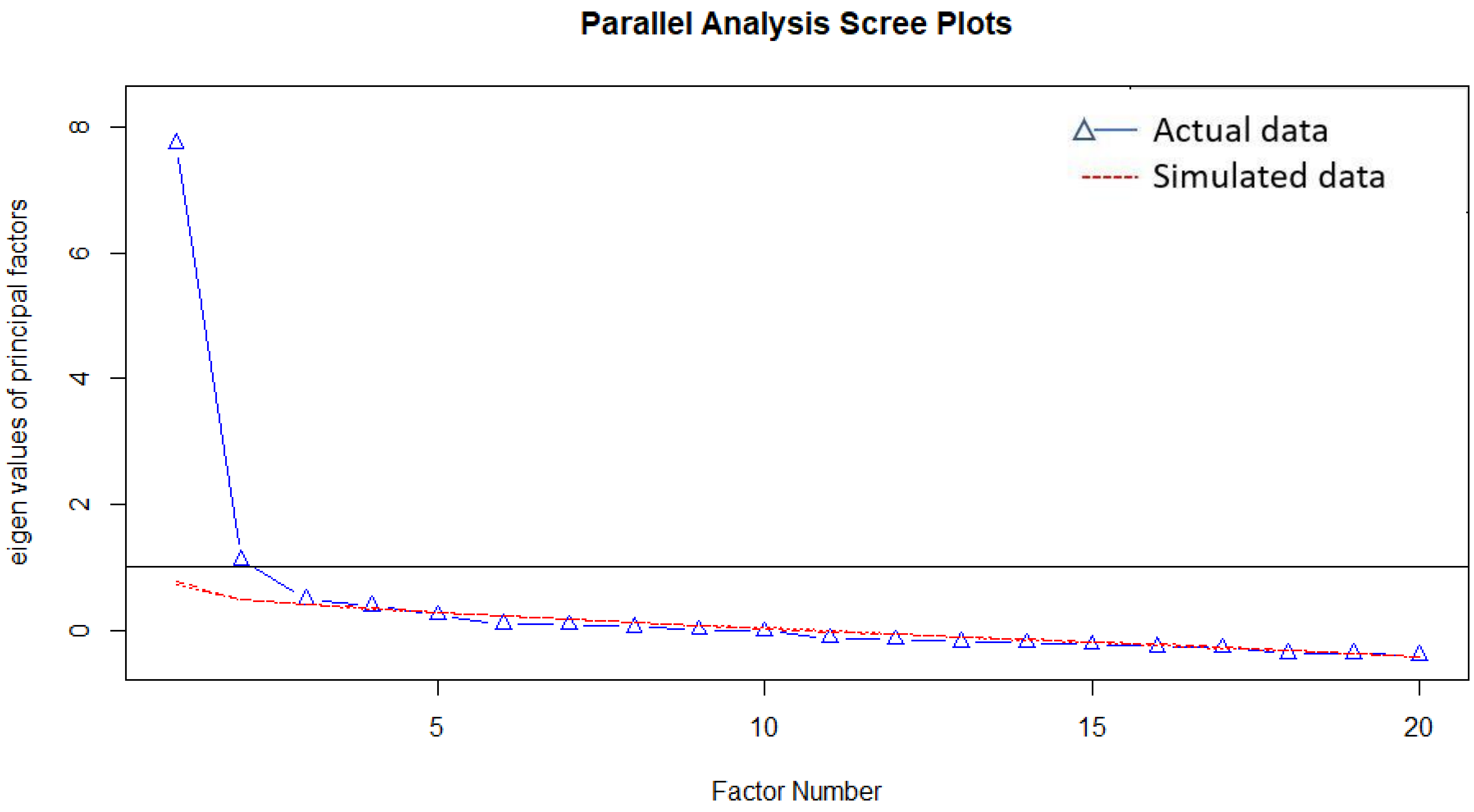

Figure 3. Scree plot following parallel analysis of the **postnatal** scale.
